# Do we care? Reporting of genetic diagnoses in multidisciplinary intellectual disability care: a retrospective chart review

**DOI:** 10.1186/s13023-024-03323-6

**Published:** 2024-09-16

**Authors:** Annelieke R. Müller, Erik Boot, Stijn B. Notermans, Carlo Schuengel, Lidewij Henneman, Martina C. Cornel, Mieke M. van Haelst, Mariëlle Alders, Clara D. M. van Karnebeek, Bas Bijl, Frits A. Wijburg, Agnies M. van Eeghen

**Affiliations:** 1 Department of Pediatrics, Emma Children’s Hospital, Amsterdam Gastroenterology Endocrinology Metabolism, Amsterdam UMC Location University of Amsterdam, Amsterdam, The Netherlands; 2 Emma Center for Personalized Medicine, Amsterdam UMC Location University of Amsterdam, Amsterdam, The Netherlands; 3 Amsterdam Public Health Research Institute, Amsterdam, The Netherlands; 4 Advisium, ‘S Heeren Loo, Amersfoort, The Netherlands; 5 The Dalglish Family 22Q Clinic, Toronto, ON Canada; 6 Department of Psychiatry and Neuropsychology, Maastricht University, Maastricht, The Netherlands; 7 Section of Clinical Child and Family Studies, Vrije Universiteit Amsterdam, Amsterdam, The Netherlands; 8 Department of Human Genetics, Amsterdam UMC Location Vrije Universiteit, Amsterdam, The Netherlands; 9 Amsterdam Reproduction and Development Research Institute, Amsterdam, The Netherlands; 10 Department of Human Genetics, Amsterdam UMC Location University of Amsterdam, Amsterdam, The Netherlands

**Keywords:** Intellectual disability, Genetic diagnosis, Diagnostics, Genetic testing, Behavioral phenotype, Multidisciplinary care, Care files, Psychodiagnostics, Caregivers, Personalized medicine

## Abstract

**Background:**

Advances in understanding the etiology of intellectual disability (ID) has led to insights in potential (targeted) treatments and personalized care. Implications of ID on health are often complex and require a multidisciplinary approach. The aim was to investigate the reporting of genetic diagnoses in multidisciplinary ID care and to identify associated clinical and demographic factors.

**Methods:**

A retrospective chart review was performed on a randomly selected sample of individuals (n = 380) of a large ID care organization in the Netherlands. Data on genetic etiology, including genetic testing and diagnoses, and clinical and demographic characteristics were collected from files held by multidisciplinary team members.

**Results:**

Reports on genetic etiology were available in 40% of the study sample (n = 151), with a genetic diagnosis recorded in 34% (n = 51), which is 13% of the total sample. In those with reported genetic diagnoses, this was reported in 90% of medical, 39% of psychodiagnostic, and 75% of professional caregivers’ files. Older age, mild ID, and the legal representative not being a family member were associated with less reported information on genetic etiology.

**Conclusions:**

This study revealed that genetic diagnoses were often not reported in ID care files. Recommendations were formulated to reduce delay in diagnosis, and enable personalized care for individuals with ID.

**Supplementary Information:**

The online version contains supplementary material available at 10.1186/s13023-024-03323-6.

## Background

About 1–3% of the population is affected with intellectual disability (ID) [1], which is characterized by substantial limitations in both intellectual functioning and adaptive behavior, originating during the developmental period [2]. Due to rapid technological advances, a genetic diagnosis can be identified in up to 50% of individuals with ID, although estimates of the diagnostic yield vary considerably across studies [3, 4]. Currently, more than 1500 monogenic causes of ID are known in addition to other causes like copy number variations (CNVs) [5]. Such genetic neurodevelopmental disorders and neurometabolic disorders often manifest with complex and variable multiorgan comorbidity. As a result, many different healthcare providers (HCPs) are usually involved in multidisciplinary care, including physicians, psychologists, and professional caregivers.

Knowing the cause of ID provides information about associated somatic and neuropsychiatric manifestations and may lead to targets for prognosis, screening, prevention, monitoring and treatment [6]. Moreover, it may result in increased life expectancy for those affected. Together with improved genetic diagnostics, targeted treatments and disorder-specific care are increasingly available [7, 8], allowing for personalized care, which is the implementation of etiology-driven health monitoring and treatments [9]. Disorder-specific care is illustrated by anticipatory care planning for individuals with Down syndrome who eventually all show neuropathological changes of Alzheimer’s disease by the age of forty [10]. Research has mainly focused on pediatric ID, although a diagnosis may provide benefits for adults too. More knowledge of complex neuropsychiatric manifestations, the greatest burden of most rare genetic neurodevelopmental disorders [11, 12], can improve targeted neuropsychological examination, psychoeducation, and behavioral interventions [13, 14]. Disorder-specific guidelines are increasingly available, providing recommendations for medical, social, psychiatric and behavioral care [15].

Although a genetic diagnosis may thus provide important benefits for affected individuals and their families, it is unknown to what extent genetic diagnoses, including information on phenotype and management, are integrated into multidisciplinary ID care.

We therefore evaluated the integration of personalized care across disciplines in ID care to identify care gaps and targets for improvements. The primary objective was to investigate the reporting of information on genetic etiology, including both genetic testing and genetic diagnoses in medical files, files used by psychologists and behavioral experts and therapists, and files used by professional caregivers. A secondary objective was to examine how often, at what time and how detailed, information on the genetic diagnosis was available in these files, and to investigate associated clinical and demographic factors to assess the integration of genetic diagnoses into clinical multidisciplinary care.

## Methods

### Study design and setting

This was a retrospective chart review at ‘s Heeren Loo, the largest care organization for ID in the Netherlands. We systematically recorded data on genetic etiology, including genetic testing and diagnosis, and clinical and demographic characteristics collected from files held by multidisciplinary team members involved.

### Care systems and study population

**Fig. 1:**
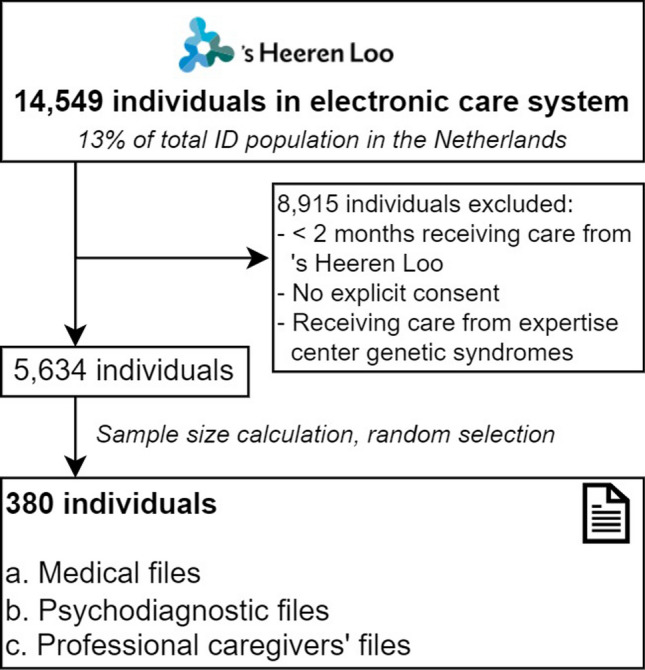
Flow diagram depicting the selection procedure of individuals eligible for electronic care file search

Individuals receiving support or care from ‘s Heeren Loo are registered in the electronic care system (Fig. 1). The system is used by all involved HCPs including behavioral scientists, to report on paramedical care, supported living, and other support. It also includes information about legal representatives. For medical care by general practitioners and ID physicians, another electronic care system is widely used.

Individuals who were registered in the electronic care system and received at least two months of support or care from ‘s Heeren Loo prior to data extraction were included in the study, unless there was no consent for using their data for research. Individuals who visited an expertise center genetic syndromes at ‘s Heeren Loo were also excluded from analyses, as a genetic diagnosis is a prerequisite for receiving care by this center.

### Procedures

The electronic care system of ‘s Heeren Loo contained data on 14,549 individuals (accessed at September 6, 2021), which is approximately 13% of the total ID population in the Netherlands [16]. Of these, around 6,200 individuals live within sheltered care facilities of the organization. The sample size for this study was based on a population size of 14,549 individuals with a standard deviation of 50%, a sampling error of 5%, and confidence interval of 95%, yielding a required sample size of ≥ 374 individuals. We randomly selected 380 individuals who met our inclusion criteria.

Electronic care files were searched for the following demographic information including type of support and care: age, sex, whether the individual had a legal representative and their relationship to the individual (e.g., family member or non-family member such as professional or friends), ID physician involvement, living situation and whether there was medical care on site. Files were reviewed to assess what information was provided on the following clinical information: severity of ID based on clinical assessments, intelligence tests and adaptive behavior assessments [2], genetic diagnosis, whether genetic diagnostics were performed, details on genetic test results if applicable, and at what age, referred by whom, total amount of genetic tests (until diagnosis), type of etiological (genetic or metabolic) test performed, the year in which it was performed, test results including classification of pathogenicity and genetic diagnosis causing the ID. A diagnosis was considered confirmed if a genetic test was performed and the specific diagnosis was confirmed with a letter from a clinical geneticist or with an available genetic test result. If no such report was available, it was assessed whether the genetic diagnosis was likely, based on available information and expert opinion (MvH, MA, AM, SN, AvE). Furthermore, given that separate files are used by specific HCPs, it was noted whether (1) medical files, (2) psychodiagnostic files used by psychologists, behavioral experts and therapists, and/or (3) files used by professional caregivers including daily care records and individual support plans were available and whether the information on genetic etiology was mentioned in these files (Fig. 1). Also, it was noted whether the involved physician was ID physician or general practitioner.

### Data analyses

Demographic and clinical characteristics were described and it was examined to what extent information on genetic etiology was available in medical files, psychodiagnostic files, and files used by professional caregivers. If a genetic cause for ID was reported in any file, it was examined whether this information was also available in files from the other disciplines as well. Independent t-tests, analyses of variance (ANOVAs), and chi-squared tests were performed to investigate whether demographic and clinical variables were associated with availability of information on genetic etiology. These variables included medical care on site, ID physician part of care team, living situation, legal representative, age, sex, and level of ID. Mann–Whitney U tests, Kruskal–Wallis or Fisher’s exact tests were used when assumptions for parametric analyses were not met. Post hoc analysis was performed using cell-wise adjusted standardized residual analysis with a Bonferroni adjusted α. A logistic regression analysis was performed to ascertain the relative effects of the associated variables on the likelihood that individuals have information on genetic etiology reported, chosen from their statistical significance on bivariate analyses. As for the legal representative variable, those with a family or non-family member as legal representative were included in the model, excluding those without a (reported) legal representative to reduce possible bias or multicollinearity. Cochran’s Q test was used to examine to what extent genetic test results were available or recorded in either medical files, psychodiagnostic files or files used by caregivers. Statistical analyses were performed using SPSS version 28.0 (SPSS Inc., Chicago, IL, USA) with a two-sided significance level of 5%.

## Results

We included 380 individuals at a median age of 46 (interquartile range 31; range 9–95) years old. Demographic and clinical characteristics are presented in Table 1.

**Table 1:** Demographic and clinical characteristics of the study sample

	Total study sample		Information available on genetic etiology	
	N = 380		N = 151	
	N	%	N	%
Demographics				
Age				
< 18 years	19	5.0	9	6.0
≥ 18 years	361	95.0	142	94.0
Sex, female	180	47.4	70	46.4
Legal representative				
None	45	11.8	10	6.6
Family member	249	65.5	118	78.1
Professional/other	86	22.6	23	15.2
ID physician involved	253	66.6	102	67.5
24 h/day care	313	82.3	121	80.1
Clinical				
Severity of ID^a^				
Mild	110	28.9	22	14.8
Moderate	130	34.2	56	37.6
Severe	97	25.5	54	36.2
Profound	41	10.8	17	11.4

*ID* Intellectual disability

^a^Severity of ID was unknown in two individuals

### Genetic diagnoses in multidisciplinary ID care

**Fig. 2:**
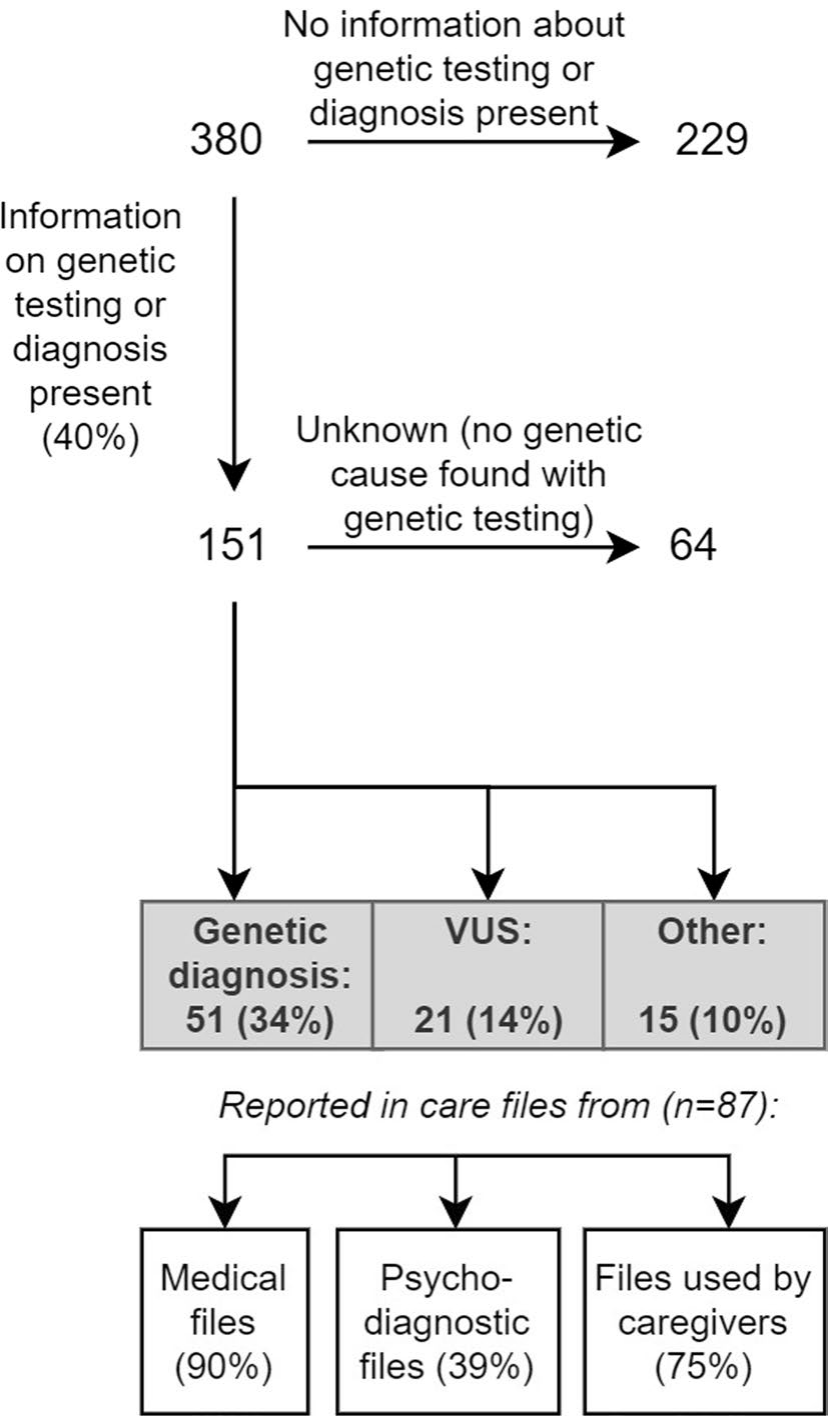
Flow diagram of sample with information on genetic etiology, according to files from different disciplines. ‘Other’ includes clinical diagnosis or genetic variants mentioned in care files as cause of intellectual disability, although insufficiently or incorrectly described to be considered as a genetic diagnosis. *VUS* Variant of uncertain significance

**Fig. 3:**
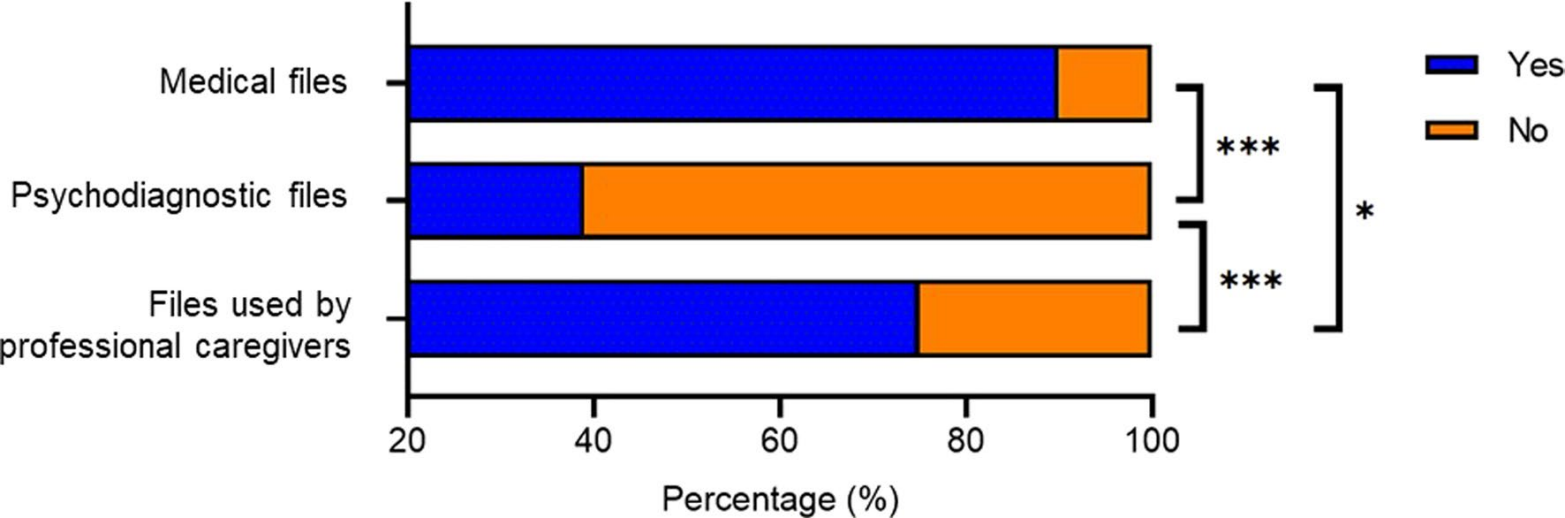
Proportion of information on genetic etiology reported in care files from different disciplines. Information on genetic etiology, including genetic diagnoses, variants of uncertain significance, and other (N = 87), reported (blue) and missing (orange) in medical files, psychodiagnostic files, or files used by professional caregivers. * indicates *p* ≤ 0.01; *** indicates *p* ≤ 0.001

Of the total study sample, information on genetic etiology was reported in the electronic care system of 151 individuals (40%) (Fig. 2). If information was recorded, most often it concerned negative test results (64/151), followed by a genetic diagnosis (51/151), variants of uncertain significance (VUS) (21/151), and clinical diagnosis or genetic variants mentioned as cause of ID although insufficiently or incorrectly described to be considered a genetic diagnosis (15/151). Particularly, VUS or genetic variants not considered a genetic diagnosis were mentioned in psychodiagnostic files or files used by professional caregivers as cause of the ID. In addition, for some individuals, the information on genetic etiology was reported as genetic diagnosis in psychodiagnostic files or files used by professional caregivers, while it was reported as VUS in medical files. The information on reported genetic cause of ID was reported in the different care files used by physicians (90%), psychologists (39%), and professional caregivers (75%) (Fig. 3). Cochran’s Q test revealed a statistically significant difference in the proportion of information reported in the care files on genetic cause of ID across the three types of care files (*X*^2^(2, *N* = 87) = 68.698, *p* < 0.001), with physicians reporting more often compared to psychologists (*p* < 0.001), and professional caregivers (*p* = 0.025), and professional caregivers reporting more compared to psychologists (*p* < 0.001).

### Factors associated with availability of information on genetic etiology

Significant associations were found between presence of information on genetic etiology and age (r = − 0.16, *p* = 0.002), the severity of ID (*X*^2^ (3, *N* = 378) = 28.898, *p* < 0.001), and the type of relationship with the legal representative (*X*^2^ (2, *N* = 380) = 17.323, *p* < 0.001) [see Additional file 1]. Post hoc analysis revealed that individuals with moderate and severe ID were more likely to have information in their files reporting on genetic etiology compared to individuals with mild ID (*p* < 0.001). Individuals with a family member as a legal representative were more likely to have this information reported compared to those without a legal representative or those with a non-family member as legal representative (*p* < 0.001). No significant associations were found between information on genetic etiology and sex, location of receiving care, and presence of medical care on site.

The logistic regression model predicting the effects of age, level of ID, and legal representative on the likelihood that individuals have information on genetic etiology reported in any file, was statistically significant (*X*^2^(5) = 48.367, *p* < 0.001), explaining 18.2% (Nagelkerke R^2^) of the variance in presence of information on genetic etiology and correctly classified 69.1% of cases (Table 2). Increasing age was associated with a decreased likelihood of reporting information on genetic etiology (odds ratio [OR] = 0.971, *p* < 0.001). Individuals with a moderate, severe or profound ID were respectively 3.36, 6.32, and 4.02 times more likely to have reported information on genetic etiology compared to individuals with a mild ID. Individuals with a family member as legal representative were 2.17 times more likely to have reported information on genetic etiology compared to those with a legal representative other than a family member.

**Table 2:** Factors associated with availability of information on genetic etiology in all files (N = 335)

Variable	B	OR	95% CI of OR		*p*
			Lower	Upper	
Age	− 0.029	0.971	0.958	0.985	< 0.001
Level of ID					
Mild	0^a^	0^a^	–	–	–
Moderate	1.211	3.36	1.663	6.779	< 0.001
Severe	1.844	6.32	2.984	13.381	< 0.001
Profound	1.391	4.02	1.595	10.123	0.003
Legal representative^b^					
Professional/other	0^a^	0^a^	–	–	–
Family member	0.776	2.17	1.215	3.882	0.009

*CI* confidence interval

^a^This group was designated as the reference category

^b^Those without a (reported) legal representative were excluded from regression analyses (N = 45)

### Reported diagnostics

Genetic testing was reported for 141 individuals: 82 (58.2%) received a test once, 33 (23.4%) twice, 15 (10.6%) three times, 8 (5.7%) four times, and 3 (2.1%) five times, with a total of 248 tests (Table 3). Metabolic testing additional to genetic testing was reported for eight (5.7%) individuals with none having positive metabolic test results, although specification on type of metabolic test was lacking. Mean age at genetic testing was 27.1 (SD 17.8) years old, with information missing for 7 cases. Karyotyping was reported most frequently (n = 73) followed by Fragile X syndrome testing (n = 49). In total, 51 individuals were reported to have a genetic diagnosis associated with ID, with genetic test results only available for 19 [see Additional file 2]. Twenty-one individuals (13.9%) were reported to have a VUS, and in 15 individuals (10.0%) a clinical diagnosis or genetic variant was mentioned by the care providers as cause of the ID, but insufficiently or incorrectly described in the absence of a letter of a clinical geneticist.

**Table 3:** Variables for whom a genetic diagnosis and/or genetic testing results were reported in files (N = 151)

Variables	N	%
Genetic testing reported	141	93.4
First genetic test^a^		
< 18 years	57	40.4
≥ 18 years	74	52.5
Not reported	10	7.1
Last time referred for genetic counseling to geneticist by		
Intellectual disability physician	38	25.2
Pediatrician	14	9.3
General practitioner	9	6.0
Other medical specialist	4	2.6
Not reported	86	57.0

Of those individuals with genetic test results reported, 57 (40.4%) had their first genetic test during childhood and 74 (52.5%) individuals during adulthood. Individuals who had their first genetic test in childhood received significantly more genetic diagnoses (*X*^2^ (1, *N* = 141) = 10.137, *p* = 0.001). There was no significant difference in ID severity between individuals who had their first genetic test during childhood or adulthood (*X*^2^ (3, *N* = 140) = 7.434, *p* = 0.059).

### Reported genetic testing over the years

**Fig. 4:**
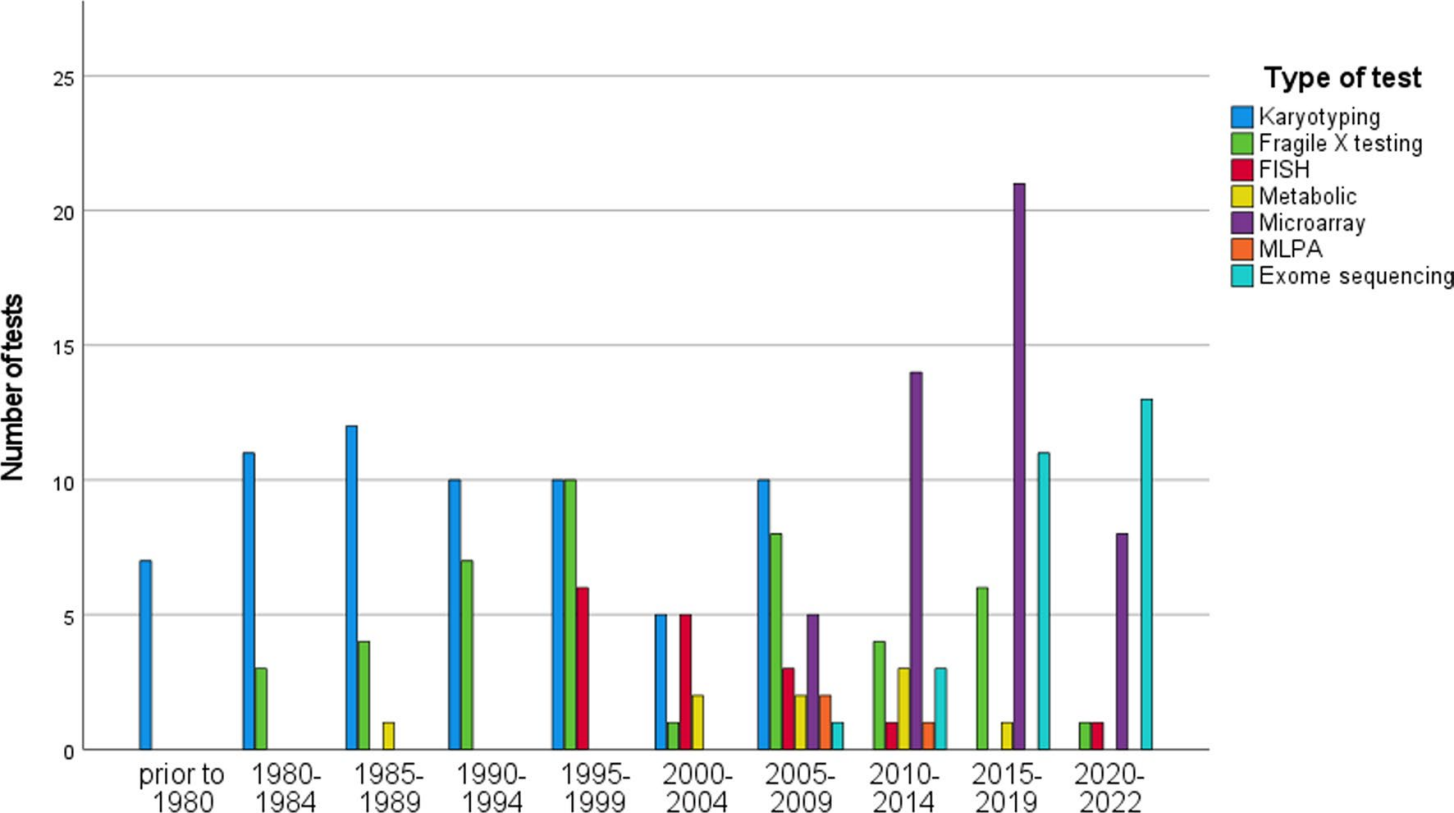
Evolution of different types of tests performed over the years, as reported in files. This information was based on files of 141 individuals with intellectual disability. *FISH* Fluorescent in-situ hybridization, *ID* Intellectual disability, *MLPA* Multiplex ligation-dependent probe amplification

Over the years, the frequency and types of genetic tests changed (Table 4; Fig. 4). Before 2005, mainly karyotyping, fluorescent in-situ hybridization (FISH) and Fragile X testing were performed. From 2005, microarrays were reported. Exome sequencing within this population was reported since 2013, with the exception of one reported in 2008 (possibly incorrect, considering the advent of this technique).

**Table 4:** Types of tests reported in medical care files of 141 individuals with ID

Type of test	Number of tests		Confirmed diagnosis		Age at testing
	N	%	N	%	Mean ± SD
Karyotyping	66	26.6	15	29.4	19.1 ± 17.2
Microarray	51	20.6	7	13.7	28.5 ± 17.9
Fragile X testing	49	19.8	6	11.8	28.3 ± 18.3
FISH	13	5.2	5	9.8	25.0 ± 16.4
Exome sequencing	28	11.3	5	9.8	29.4 ± 14.6
MLPA	3	1.2	1	2.0	43.0 ± 2.6
Metabolic	8	3.2	0	0.0	27.3 ± 13.0
Unknown	30	12.1	12	23.5	29.0 ± 21.1
Total	248	100	51	100	

*FISH* Fluorescent in-situ hybridization, *ID* Intellectual disability, *MLPA* Multiplex ligation-dependent probe amplification

Before 1995, the majority of genetic tests were reported to be performed in children and young adults, while older adults were incidentally tested. In 2004, the first individual over the age of 60 years was tested, and since then 14 more. In the last two decades, older individuals have been increasingly tested according to care files, and the overall number of genetic tests reported increased over time.

Of 51 individuals who were reported to have received microarray analysis, 7 (13.7%) received a diagnosis, and another 18 (35.3%) received additional exome sequencing analysis, yielding 3 (6%) additional diagnoses. Of the total number of individuals who received exome sequencing (N = 28), 5 (17.9%) had a genetic diagnosis and 11 (39.3%) a VUS.

## Discussion

This retrospective chart review shows variable reporting of genetic diagnoses by different types of care providers, revealing a gap for optimal personalized care for individuals with ID. All care providers involved in the care must be aware of the genetic diagnosis. Electronic care file systems accessible to all care providers with harmonized coding will ensure consistent reporting of genetic diagnoses in ID care. Significant associations were found between availability of information on genetic etiology (including genetic testing and diagnoses) in care files and individual’s age, level of ID, and the legal representative’s relationship to the individual.

### Reporting genetic diagnoses in multidisciplinary ID care

Genetic causes of the ID were reported in 90% of medical files, 39% of psychodiagnostic files, and 75% of files used by professional caregivers, although VUS or incorrectly described genetic information were mentioned as cause of the ID in some of these different care files, thus misinterpreting these ambiguous or uncertain genetic findings. This suggests that adequate documentation of a genetic diagnosis is not standard part of multidisciplinary ID care. Barriers for reporting genetic diagnoses to explain the ID in multidisciplinary care have been found to include lack of awareness of potential benefits, lack of communication and harmonization of coding, and difficulty interpreting the results [17]. It may imply that many individuals with ID miss out on disorder-specific medical care, psychological care, and support. This is unfortunate, as a genetic diagnosis can provide detailed information on the prognosis of the disorder, associated somatic and neuropsychiatric manifestations, and targets for prevention, treatment, and management. It is also important for unaffected family members who might be at risk of passing on a genetic condition to their future children. As it may have impact on all life domains, awareness of all types of HCPs involved is necessitated to improve care [18].

Surprisingly, not all physicians who were involved in the care team had a reported genetic diagnosis in their medical files, while it was mentioned in one of the other care files, although not verified due to absence of a letter of a clinical geneticist. Coordinating physicians should have direct access to the genetic test results, which means they could inform and update the multidisciplinary team to enable personalized and disorder-specific care, and refer to expert centers where available. From a medical perspective, this may include each body system, including epilepsy management [19], tumor screening [15], prevention for sensitivity to obesity [20] and movement disorders [21], for which a dietician, physiotherapist, or occupational therapist should be involved as well. Without knowledge on the etiology, physicians will not identify and refer candidates who may benefit from disorder-specific care, including condition-specific guidelines or targeted treatments, such as indicated by the Treatable ID (Web) App [7].

Psychologists and behavioral therapists have a major role with regard to timely consultation of other experts, psychoeducation of care teams and families, treating complex behavioral manifestations, and providing information on appropriate behavioral interventions, guidance and mentoring, preventing frustration, crisis and overmedication [13]. Increasing knowledge on syndrome-specific behavioral manifestations is available [22, 23].

Caregivers are often the first to detect possible disorder-specific manifestations. Understanding the etiology of somatic and behavioral manifestations is of great importance for early signaling and to respond adequately. Especially in complex situations, comprehending the cause and support needs contributes to establishment of a shared concept and vision and multidisciplinary management. This may increase empowerment and anticipatory care planning.

For legal representatives who take care decisions for the affected individual, we found that individuals with close proximity of their legal representative such as first- and second-degree family member appeared to be more likely to receive genetic testing compared to those with a legal representative other than direct family, such as a professional. Family may be more engaged in health management, and may also directly benefit from a diagnosis by better understanding and acceptance, information on recurrence risks and prenatal diagnostic options, and prognostic value about whether someone could still live at home or need professional caregivers. Additional benefits for the affected individual or family members include supportive care, special education or tools, access to expertise centers and (peer) support groups, and financial and emotional support [24–26].

### Diagnostic care gap

If current local and international (pediatric) guidelines were followed, one would expect that most individuals with ID had been referred to a clinical geneticist [27]. However, in our study, only in 40% of individuals with ID reports on genetic etiology were available in care files. A genetic diagnosis was identified in 34% of these individuals (which is 13% of the total sample), although official results were often not available in the electronic care file system. These results on current clinical practice demonstrate that genetic testing is underutilized, comparable to a previous study in Scotland that reported 41% of individuals with ID had genetic testing with a reported genetic cause for ID in 6% [28].

We found that more severe levels of ID, lower age, and close proximity of the legal representative’s relationship to the individual were associated with increased reporting of information on genetic etiology, indicating disparities in access to genetic testing. Notably, genetic testing in individuals with ID might differ throughout countries and cultures. Since European countries such as the Netherlands have a high standard with regard to easy and paid access to medical care, the care gap may be expected to be even greater in other countries. In the Netherlands, physicians can order specific genetic testing, such as microarrays or gene panels. In addition to paying a monthly premium for health insurance, there is an annual deductible that individuals must meet before their insurance covers certain healthcare costs. Genetic testing falls under this own contribution, but when it is met (which is often the case with this patient population), these costs are covered by the health insurance.

Factors associated with availability of information on genetic etiology in files may indicate both a reporting and diagnostic care gap. Individuals with a higher age appeared to be less likely to have reported information on receiving genetic testing and diagnosis. Our results confirm previous findings that a genetic diagnosis is lacking in many adults [29, 30], possibly including reasons such as less relevance to parents of affected adults in terms of recurrence risk. On the other hand, one could argue that older individuals would have a higher chance of having information on genetic etiology available, as they have had more time to be tested. As the largest population comprises adults, of which the majority did not receive a genetic diagnosis, these might thus miss out on personalized care.

Furthermore, individuals with mild ID appeared to be less likely to have reported information on genetic testing and diagnosis compared to those with moderate, severe or profound ID, possibly due to HCPs being less likely to consider genetic testing for mild ID and due to the fact that there seems to be more knowledge about the genetic causes associated with greater disability. On the other hand, many genetic syndromes show great heterogeneity regarding the level of intellectual functioning, with individuals with no or mild ID possibly being underrepresented and missing a diagnosis. Those with mild ID might thus more frequently miss out on disorder-specific care and interventions, underlining the need of awareness and guidelines.

Several barriers for the integration of genetic diagnoses into ID care may exist, including lack of parents for trio exome sequencing, financial issues, and lack of motivation by HCPs [31]. Practical barriers mentioned by physicians in previous research include lack of capacity or unavailability of consent by caregivers, burden and distress, unacknowledging benefits and skepticism about clinical utility especially in adults, and a lack of training resulting in difficulty interpreting and explaining genetic test [17, 28].

### Recommendations and future directions

To overcome barriers and contributors to care gaps to identify individuals with ID at risk for underdiagnosis and undertreatment of genetic disorders, recommendations are provided in Table 5. Care organizations should connect with regional clinical genetic centers to reduce the referral threshold and diagnostic delay, for broader implementation of frontline tests (e.g., microarray and exome sequencing), for reassessment of whether additional testing might be of diagnostic benefit, and for reanalysis of VUS in genes for which functional tests are available. This may include episignatures which could provide conclusive findings for around 70 known ID syndromes as these have been considered highly sensitive, and specific DNA methylation biomarkers [32]. Education for affected individuals, families, caregivers, (professional) legal representatives, and all types of HCPs on both the importance of genetic testing and the genetic diagnosis may increase awareness and empowerment, and improve quality of multidisciplinary personalized care [33–35]. Adult care, which has usually been variable and fragmented, has greatly improved, advocating for holistic expert care worldwide.

**Table 5:** Recommendations to overcome barriers to care gaps for individuals with ID at risk of underdiagnosis

	Barriers	Recommendations
	Limited access to genetic testing	Develop, update, and implement (international) protocols and guidelines for genetic testing (especially for adult ID)
		Stimulate close collaborations between (academic) clinical genetic centers and physicians involved in ID care
		Facilitate periodic consultations (live or virtual) with a clinical geneticist at the ID facility (e.g., for pre- and post-test counseling, and treatment options)
		Reduce practical barriers to testing (e.g., train HCPs for genetic diagnostics in regional care networks)
		Reduce (patient) burden of testing (e.g., using saliva samples (when suitable for the intended test) instead of blood samples)
		Implement protocol for periodic reanalysis of variants of uncertain significance and repeat genetic testing when no diagnosis was identified
		Increase transparency on insurance reimbursement of genetic testing if applicable
		Develop accessible and comprehensible information on somatic and neuropsychiatric manifestations of genomic variants for all HCPs involved
		Increase understanding of the importance of recurrence risks and prenatal diagnostics for affected individual or (healthy) family members; refer to clinical geneticist in case of unknown diagnoses
		Increase awareness of the implications of possible negative attitudes towards genetic testing among affected individuals, carers and HCPs (e.g., perceived low yield, insurance problems, fear of stigmatization)
	Limited reporting; coding and harmonization	Establish protocols to harmonize coding and facilitation of ICT systems for communication between HCPs, also to ensure continuity of care

	Contributors to decreased reporting of genetic diagnoses	Recommendations
	Type of HCP	Provide education and information to understand importance of a genetic diagnosis for care, for physicians, psychologists, and caregivers
		Improve availability of, and access to, physicians with knowledge on genetic disorders and associated manifestations
		Clarify the role of coordinating physician for referring for (re-)evaluation of genetic diagnosis and inform other care providers
		Implement genetic etiology as standardized part of reporting in medical files and individual support plans in individuals with ID
	Age	Explicitly include adults with ID in guidelines for genetic testing
		Ensure inclusion of genetic test results when transferring individuals with ID to other HCPs, for example in the transition from pediatric to adult care or from parental home to residential care
		Ensure access to all medical information by the coordinating local physician, especially when transitioning to adult care
	Level of ID	Improve awareness of the benefits of genetic testing in care providers of individuals with mild ID and/or limited somatic comorbidity, including guidelines for indications for genetic testing in individuals with for borderline intelligence, e.g., with suspect somatic, psychiatric or neurologic comorbidity
	Legal representative	Increase awareness on care gap of absence of a family member as legal representative, and education for caregivers

Recommendations are provided by the authors to enable disorder-specific personalized care and empowerment with regard to diagnostics. *HCP* Healthcare provider, *ID* Intellectual disability

Pediatric guidelines should be extended to adults, since implications of a diagnosis are important for the adult population as well. Individuals with no or borderline ID could also have a genetic disorder, as many genetic syndromes show a great heterogeneity within the disorder, which also requires special attention in psychiatric care. An update on current diagnostic guidelines including genetic testing and counseling in psychiatry has been proposed [36]. Awareness on factors that contribute to a diagnostic care gap should be increased to prevent them from missing out on personalized treatments, management and screening.

Furthermore, electronic care file systems should be improved for this patient population. Protocols should be established for harmonized coding of genetic diagnoses such as using OMIM and ORPHA code. Communication between HCPs should be facilitated by ICT systems, ensuring continuity, transferability and linkage to central relevant sites.

Expertise centers for rare diseases should (inter)nationally assemble, like the European Reference Network (ERN-)ITHACA (https://ern-ithaca.eu/), as these disorders collectively affect many individuals worldwide. These networks contribute to disorder-specific knowledge, including natural history, updating information with regard to the disorder and treatment options, setting up registries, and guidelines, and implementing these in national and regional care networks. This should be performed together with affected individuals and representatives, to also ensure availability of other resources for specific disorders, such as (peer) support groups [28].

Future research is necessary to examine why knowledge of genetic testing has not been fully implemented, to further identify barriers to personalized care. For instance, as the natural history of (ultra)rare disorders is often unknown, health care providers may question whether a genetic diagnosis really results in better care at present [37]. However, positive experiences in care and benefits for individuals should inspire all to enable and improve disorder-specific care.

### Strengths and limitations

This is the first study that elaborately investigated the integration of genetic diagnoses into multidisciplinary ID care in a large sample based on a sample size calculation. However, representativeness of the data may be affected by the consent procedure: bias might have occurred, since for individuals living within sheltered care facilities of the organization a standard questionnaire is included for providing consent. Selection bias with regard to symptoms, dysmorphisms, suspicion of syndromes, and comorbid features towards those who received genetic testing was not investigated. Moreover, negative genetic test results might not have been documented or (non-digital) information might be lost by switching care facilities or care providers, such as in the transition from pediatric to adult care. Due to privacy regulations, genetic results are usually only sent to the referring physician. Also, letters from clinical geneticists were often lacking in medical files, and genetic findings were sometimes unclear or incorrectly described by the care provider. Additional genetic variants of clinical relevance were not reported, although these have also been identified in genetic syndromes and ID [38]. As this was a retrospective study in a clinical setting, we could not examine the diagnostic yield of genetic testing [39].

We encountered difficulties related to inconsistent use of terminology and the lack of a uniform registration in the electronic care system where diagnoses could be found. Genetic disorders are often known by multiple names, possibly resulting in confusion and illustrating the importance of education amongst care providers.

## Conclusions

This study showed variable reporting of genetic diagnoses in multidisciplinary ID care files. Type of reporting care provider, milder levels of ID, a higher age, and no family member as legal representative were associated with less reporting and may consequently limit personalized multidisciplinary care. Due to fast advances in the field of diagnostics and targeted interventions, closer collaboration between academia and care organizations is necessary to improve integration of knowledge into daily multidisciplinary practice. Increased genetic testing and adequate reporting of test results over life may improve patient support, outcomes, and allow targeted therapies and surveillance.

## Supplementary Information

Additional file1: Factors associated with availability of information on genetic etiology in care files using univariate analyses (N = 380).

Additional file 2: Genetic diagnoses as reported in the electronic care system.

## Data Availability

The datasets generated and analyzed during the current study are not publicly available due to privacy reasons and sensitive information, but are available from the corresponding author on reasonable request.
