# Supplementary material for: Do we care? Reporting of genetic diagnoses in multidisciplinary intellectual disability care: a retrospective chart review
Source: Orphanet J Rare Dis. 2024 Sep 16;19:346. doi: 10.1186/s13023-024-03323-6 (PMC11403852; doi:10.1186/s13023-024-03323-6)
Supplement: Supplementary file 1 — Additional file1: Factors associated with availability of information on genetic etiology in care files using univariate analyses (N = 380). [file 13023_2024_3323_MOESM1_ESM.pdf]

Additional file 1

Factors associated with availability of information on genetic etiology in care files using univariate analyses ($N = 380$).

Variable	Effect size	*p*
Age	r = -0.16	.002
Sex	X^2 = 0.049	0.83
Level of ID	X^2 = 28.898	<.001
Location of receiving care	X^2 = 29.567	.08
Medical care on site	X^2 = 0.349	.55
Legal representative	X^2 = 17.323	<.001

ID, intellectual disability
