# Supplementary material for: Do we care? Reporting of genetic diagnoses in multidisciplinary intellectual disability care: a retrospective chart review
Source: Orphanet J Rare Dis. 2024 Sep 16;19:346. doi: 10.1186/s13023-024-03323-6 (PMC11403852; doi:10.1186/s13023-024-03323-6)
Supplement: Supplementary file 2 — Additional file 2: Genetic diagnoses as reported in the electronic care system. [file 13023_2024_3323_MOESM2_ESM.pdf]

Additional file 2

Genetic diagnoses as reported in the electronic care system.

Reported genetic diagnosis	OMIM	N	Genetic test results unavailable
Down syndrome	190685	15	15
Fragile X syndrome	300624	8	7
Smith-Magenis syndrome	182290; 607642	5	3
22q11.2 deletion syndrome	188400; 192430	4	1
Epileptic encephalopathy	176260	2	
Prader-Willi syndrome	176270; 615547	2	1
Pitt-Hopkins syndrome	610954	2	1
18p duplication syndrome	N.A.	1	
18p deletion syndrome	N.A.	1	
ZNF292 syndrome	619188	1	
Miller-Dieker syndrome	247200	1	
1q21.1 microdeletion syndrome	612474	1	
Trisomy 9p	N.A.	1	
PURA syndrome	616158	1	
Alpha-thalassemia-intellectual disability syndrome	141750	1	
Tuberous Sclerosis Complex	191100; 613254	1	1
Schindler disease	609241; 609242	1	1
Williams syndrome	194050	1	1
DeSanto Shinawi syndrome	616708	1	1
WDFY3-related syndrome	N.A.	1	
Total		51	32
